# Characteristics of Chitosan Films with the Bioactive Substances—Caffeine and Propolis

**DOI:** 10.3390/jfb14070358

**Published:** 2023-07-09

**Authors:** Karolina Stefanowska, Magdalena Woźniak, Anna Sip, Lucyna Mrówczyńska, Jerzy Majka, Wojciech Kozak, Renata Dobrucka, Izabela Ratajczak

**Affiliations:** 1 Department of Chemistry, Faculty of Forestry and Wood Technology, Poznan University of Life Sciences, Wojska Polskiego 75, 60625 Poznań, Poland; izabela.ratajczak@up.poznan.pl; 2 Department of Biotechnology and Food Microbiology, Faculty of Food Science and Nutrition, Poznan University of Life Sciences, Wojska Polskiego 48, 60627 Poznań, Poland; anna.sip@up.poznan.pl; 3 Department of Cell Biology, Faculty of Biology, Adam Mickiewicz University, Uniwersytetu Poznańskiego 6, 61614 Poznań, Poland; lucyna.mrowczynska@amu.edu.pl; 4 Department of Wood Science and Thermal Techniques, Faculty of Forestry and Wood Technology, Poznan University of Life Sciences, Wojska Polskiego 38/42, 60637 Poznań, Poland; jerzy.majka@up.poznan.pl; 5 Department of Industrial Products and Packaging Quality, Institute of Quality Science, Poznań University of Economics and Business, al. Niepodległości 10, 61875 Poznań, Poland; wojciech.kozak@ue.poznan.pl (W.K.); renata.dobrucka@ue.poznan.pl (R.D.)

**Keywords:** biomedical materials, chitosan, propolis, caffeine, chitosan films

## Abstract

Chitosan is a natural and biodegradable polymer with promising potential for biomedical applications. This study concerns the production of chitosan-based materials for future use in the medical industry. Bioactive substances—caffeine and ethanolic propolis extract (EEP)—were incorporated into a chitosan matrix to increase the bioactivity of the obtained films and improve their mechanical properties. Acetic and citric acids were used as solvents in the production of the chitosan-based films. The obtained materials were characterized in terms of their antibacterial and antifungal activities, as well as their mechanical properties, including tensile strength and elongation at break. Moreover, the chemical structures and surface morphologies of the films were assessed. The results showed that the solution consisting of chitosan, citric acid, caffeine, and EEP exhibited an excellent antiradical effect. The activity of this solution (99.13%) was comparable to that of the standard antioxidant Trolox (92.82%). In addition, the film obtained from this solution showed good antibacterial activity, mainly against *Escherichia coli* and *Enterococcus faecalis*. The results also revealed that the films produced with citric acid exhibited higher activity levels against pathogenic bacteria than the films obtained with acetic acid. The antimicrobial effect of the chitosan-based films could be further enhanced by adding bioactive additives such as caffeine and propolis extract. The mechanical tests showed that the solvents and additives used affected the mechanical properties of the films obtained. The film produced from chitosan and acetic acid was characterized by the highest tensile strength value (46.95 MPa) while the chitosan-based film with citric acid showed the lowest value (2.28 MPa). The addition of caffeine and propolis to the film based on chitosan with acetic acid decreased its tensile strength while in the case of the chitosan-based film with citric acid, an increase in strength was observed. The obtained results suggested that chitosan films with natural bioactive substances can be a promising alternative to the traditional materials used in the medical industry, for example, as including biodegradable wound dressings or probiotic encapsulation materials.

## 1. Introduction

In recent years, there has been a growing interest in non- or low-toxic materials from natural sources or from waste in various fields. Studies have shown that chemicals and synthetic materials can have negative impacts on the environment and human health. Due to the growing threats and greater consumer awareness, scientists and researchers have turned their attention to polysaccharides, which are polymers that occur naturally in nature [1,2].

Polysaccharides, such as chitosan, cellulose, and glycogen, are known for their biocompatibility, which means they have no negative impacts on the environment, and these materials are also biodegradable, meaning they can decompose naturally without posing a threat [1]. However, these characteristics are not the only advantages of polysaccharides. One of the main reasons for their growing popularity is the fact that they can be subjected to various chemical modifications, making it possible to adapt their structures and properties to specific applications [1,3,4]. Another advantage of polysaccharides is that they can be easily obtained from a variety of sources, such as shrimp and crab shells for chitosan, wood or biomass for cellulose, or algae for alginates. This makes them readily available and economically viable to obtain [1]. In addition, the chemical modification processes of polysaccharides allow for the introduction of different functional groups and changes in their structures, leading to materials with desirable physicochemical properties [5].

In medicine, pharmacy, and biology, the use of polysaccharides and polysaccharide-based materials is opening up new possibilities. Cellulose can serve as a carrier for various pharmaceutical compounds and as a matrix for biomaterials and tissue scaffolds [6,7]. Chitosan can be used in the production of dressings, implants, or drug carriers [1,8,9,10,11,12]. Chitosan-based materials, which exhibit unique biological and physicochemical properties, are an important group of materials used in biomedicine [13,14,15,16]. Chitosan, as a natural polysaccharide, has many advantages, such as non-toxicity, biocompatibility, biodegradability, and antimicrobial properties [17,18,19]. Chitosan is a promising candidate for use as a matrix in natural and biodegradable composite materials due to its many beneficial properties. It is a polymer derived from chitin, which is one of the most widespread biopolymers in nature. It is extracted from crustacean shells, which are a byproduct of the food industry [20]. 

One of the most prominent developing applications of chitosan materials is the production of biological dressings [1,8,21,22,23,24]. Chitosan-based dressings show the ability to stimulate wound healing through the controlled release of growth factors or antiseptics and have the ability to absorb excess fluid from a wound [21,22,23]. In addition, materials based on chitosan have been used in the manufacture of biomedical implants, as well as in tissue engineering, where they are used as scaffolds for tissue culture and regeneration [12,25]. The use of chitosan materials in tissue engineering has opened up new possibilities in regenerative and reconstructive therapies. Due to its biocompatibility, chitosan can be used as a material for creating implants such as sutures, prostheses, and tissue reconstruction components [26,27]. Moreover, chitosan provides suitable conditions for cell growth, promoting cell adhesion, proliferation, and differentiation owing to its structure and biological properties [1,25,28]. Recently, a number of scientific articles have been published discussing the use of various chitosan-based materials in drug delivery [19,29,30,31,32]. Chitosan shows the ability to absorb various biologically active substances, and because of this, it can be used to deliver drugs to specific sites in the body [30]. The use of chitosan as a drug carrier can significantly increase the effectiveness of a therapy while minimizing drug toxicity [30,32]. 

The physical, mechanical, and biological properties of chitosan-based films relate to chitosan’s parameters, including the degree of deacetylation and molecular weight, as well as its source [33,34,35,36]. According to the literature data, chitosan solutions show strong antibacterial effects, while the chitosan films obtained from the film-forming solutions lose this activity [37]. Since the antimicrobial activities of materials for biomedical applications are very important, researchers are investigating the possibility of using natural additives, such as plant extracts or essential oils and their components, to enhance the antimicrobial activities of chitosan films and achieve synergistic effects [2,36,38,39]. 

Caffeine belongs to the xanthine alkaloids and is likely the most widely consumed pharmacologically active substance by humans [40]. It is found in common beverages, including coffee, tea, and energy drinks, and in products containing chocolate or cocoa, as well as in some drugs [41]. The antioxidant properties of caffeine help protect the body from free radical damage and may reduce the risk of oxidative stress-related diseases [42]. In addition, coffee extracts show also antimicrobial activities against various pathogens, including *Streptococcus aureus*, *Listeria monocytogenes*, *Streptococcus mutans*, *E. coli,* or *Candida albicans* [43,44].

Propolis (bee glue) is a natural plant-derived complex collected by honeybees from various plants and trees [45,46]. Since ancient times, bee glue has been used in traditional medicine due to the healing properties of its extracts, such as its anti-inflammatory, antiseptic, anesthetic, and antioxidant effects [46,47,48,49]. Propolis extracts have found effective use in the treatment of wounds and burns [50,51]. In addition, propolis extracts have shown antimicrobial activities against pathogens, such as *S. aureus*, *E. coli*, *L. monocytogenes*, *Bacillus cereus,* and *Pseudomonas fluorescens* [52,53,54]. The wide range of the biological activities of propolis extracts is due to its complex chemical composition, which includes phenolic compounds, alcohols, terpenes, enzymes, vitamins, amino acids, sugars, and elements [55,56]. The literature data have shown that propolis extracts have been successfully used as additives to films based on biopolymers, including chitosan, to improve antimicrobial properties [36,57,58,59].

**Figure 1:**
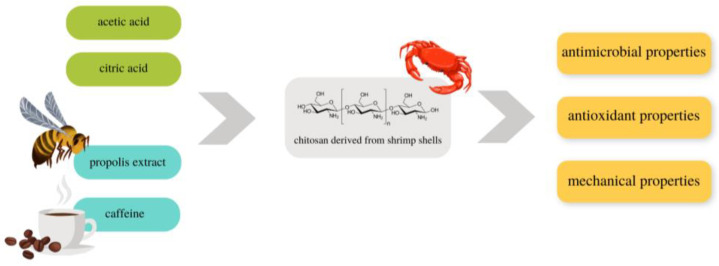
Scheme of the research.

The aim of this research was to obtain films based on shrimp-derived chitosan with the addition of the biologically active substances: ethanolic propolis extract (EEP) and caffeine. Two acids—acetic and citric—were used to prepare the film-forming solutions. The obtained films were characterized in terms of their mechanical, structural, and antimicrobial properties to assess their potential in biomedical applications as biodegradable wound dressings. The scheme of the research is presented in Figure 1.

## 2. Materials and Methods

### 2.1. Preparation of Chitosan-Based Films

Chitosan from shrimp shells (4 g) (≥75% deacetylated, molecular weight: 190,000–375,000 Da, Sigma Aldrich, Darmstadt, Germany) was dissolved in 400 mL of two solvents: 3% acetic acid solution (Avantor Performance Materials, Gliwice, Poland) and 3% citric acid solution (Avantor Performance Materials, Gliwice, Poland). After homogenization, the mixtures were poured into Petri dishes that were coated with Teflon and left to dry at room temperature.

The chitosan-based forming solutions were used to prepare the films with additives (caffeine and propolis extract). The chitosan film-forming solution (400 mL) was mixed with caffeine (Sigma Aldrich, Darmstadt, Germany) to obtain a final concentration of caffeine equal to 1% and with Tween-20 (1.5 mL) (Sigma Aldrich, Darmstadt, Germany). Once the mixture was homogenized, it was poured into Petri dishes that were lined with Teflon. The films were then dried at room temperature.

In order to prepare the chitosan film with caffeine and propolis, the chitosan–caffeine solution (400 mL) was combined with the ethanolic extract of propolis (PROP-MAD, Poznań, Poland) to obtain a final concentration of 1% propolis. Once homogenized, the solution was poured into Petri dishes that had Teflon liners, and the resulting film was left to dry at room temperature. 

Table 1 illustrates the symbols representing the 6 distinct chitosan-based film samples acquired as an outcome of the experiment.

### 2.2. Antiradical Effect of Chitosan-Based Solutions

The antiradical activities of the chitosan film-forming solutions were evaluated using a DPPH (2,2-diphenyl-1-picrylhydrazyl, Sigma Aldrich, Darmstadt, Germany) radical scavenging assay. The antioxidant properties were measured and calculated according to the procedure described in our previous paper [60]. Each solution (3 µL) was mixed with distilled water (297 µL), vortexed, combined with 0.1 mM DPPH solution (300 µL), and incubated in the dark for 30 min. The absorbance readings (A) of the solutions were measured at λ = 517 nm by a BioMate^TM^ 160 UV-visible spectrophotometer (Thermo Scientific, Waltham, MA, USA). Trolox (Sigma Aldrich, Darmstadt, Germany) was used as a reference antioxidant. Each sample was prepared in triplicate and the results are presented herein as the samples’ antiradical activities (%).

### 2.3. Antimicrobial Activity of Chitosan-Based Films

An agar diffusion method was used for the assays of the antimicrobial activities of the chitosan-based films. In the antimicrobial activity tests, the indicators were the following pathogenic or potentially pathogenic bacteria strains: *Bacillus subtilis* (food isolate), *Enterococcus faecalis* (food isolate), Staphylococcus aureus (ATCC 25923), *Escherichia coli* (ATCC 10536), *Pseudomonas aeruginosa* (ATCC 15443), *Pseudomonas fluorescens* (food isolate), and *Salmonella enterica* (clinical isolate), as well as the following probiotic bacteria strains: *Lacticaseibacillus rhamnosus* GG (ATCC 53103), *Lactiplantibacillus plantarum* 299v (isolate from Sanprobi IBS, Poland), and *Lacticaseibacillus paracasei* (CNCM I-1572). The tests also included the following fungi strains: *Candida albicans* (fecal isolate), *Aspergillus niger* (food isolate), and *Aspergillus flavus* (food isolate). The food isolates were obtained from the Department of Food Biotechnology and Microbiology collection at Poznan University of Life Sciences. The bacteria and yeast strains were grown on TSA broth (OXOID CM 0129) for 24 h at 37 °C. The cells were collected in sterile peptone water and their solutions were adjusted to 0.5 standard McFarland turbidity (1.5 × 10^8^ CFU/mL). In order to assess the antimicrobial activity, the discs of chitosan-based films with 10 mm diameters were placed on Petri dishes with Müller Hinton agar (OXOID CM 0337) inoculated with the prepared cell suspensions (100 μL). Then, the dishes were incubated for 24 h at 37 °C. After this time, the diameters of the inhibitory zones surrounding the discs were measured in mm using a computer scanning system (MultiScaneBase v14.02). The mold cultures were carried out for 14 days at 28 °C on PDA agar (OXOID CM 0139), and then discs with diameters of 20 mm were cut out of them. These discs were placed in the centers of Petri dishes with PDA agar and the film samples. During 28 days of incubation at 28 °C, mold growth was observed. Growth inhibition was determined when there was no visible growth around the chitosan-based films.

### 2.4. Mechanical Properties of Chitosan-Based Films

The mechanical features of the chitosan-based films were determined using a testing machine (Model 5965, Intron, Burlington, MA, USA). The tests were conducted at a speed of 100 mm/min. Testing strips were cut to sizes of 1.5 cm × 10 cm. The mechanical properties, including tensile strength (TS) and elongation at break (EB), were calculated as average values from ten replicates.

### 2.5. Scanning Electron Microscopy

The surface morphologies of the chitosan-based films were determined by an Evo 40 scanning electron microscope (Zeiss, Oberkochen, Germany) operating in beam mode at 20 kV with a secondary electron detector. Before analysis, the chitosan-based films were cut to size and sprayed with gold.

### 2.6. Fourier Transform Infrared Spectroscopy

The structural analyses of the chitosan-based films were conducted by attenuated total reflectance Fourier transform infrared spectroscopy (ATR-FTIR) using a Nicolet iS5 spectrophotometer (Thermo Fisher Scientific, Waltham, MA, USA). The spectra were obtained in the range of 4000–600 cm^−1^ with a resolution of 4 cm^−1^, and a total of 32 scans were recorded.

### 2.7. Statistical Analysis

The statistical analyses included a factorial one-way ANOVA, followed by a Tukey’s honest significant difference (HSD) test with a significance level of α = 0.05. All statistical computations were conducted using TIBCO Software Inc.’s Statistica version 13.3 (Palo Alto, CA, USA).

## 3. Results

### 3.1. Antioxidant Activity of the Film-Forming Solutions

**Figure 2:**
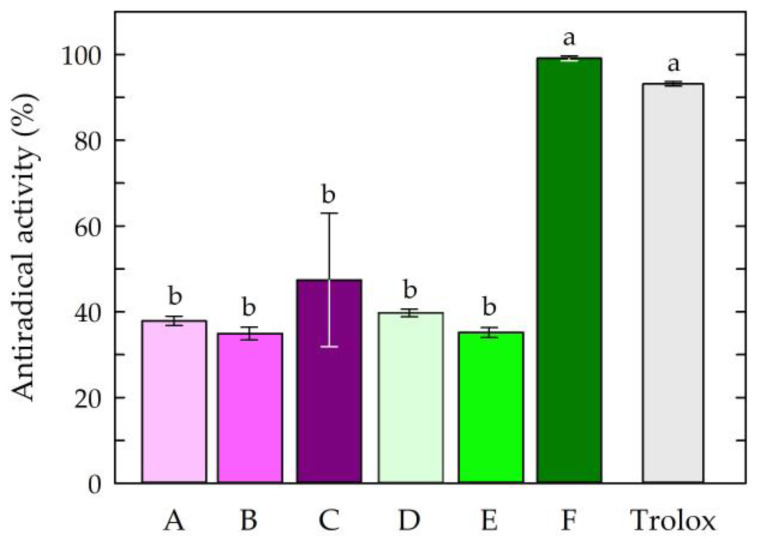
Antiradical activities of the chitosan film-forming solutions. Symbols of chitosan-based solutions (A–F) according to Table 1. The different letters above the bars in the graph indicate statistically significant differences at *p* < 0.05.

The first step of the research was to examine the antioxidant potential of the chitosan film-forming solutions. The antiradical activities of the chitosan-based solutions are presented in Figure 2.

The obtained results indicated that the solution consisting of chitosan, citric acid, caffeine, and propolis extract (solution F) showed the highest antiradical activity among all tested chitosan-based solutions. The activity of this solution (99.13%) was comparable to that of the standard antioxidant Trolox (92.82%), which was confirmed by statistical analysis. The other chitosan-based solutions exhibited similar and lower free radical scavenging capacities compared to Trolox and solution F. The results of the study showed that chitosan solutions devoid of natural active ingredients, such as propolis and caffeine, showed minor free radical scavenging ability, regardless of the type of acid used. The study showed that the use of caffeine as an additive to chitosan solutions did not statistically change the antioxidant activities of the solutions. The high antioxidant activity of the chitosan-based solution with caffeine and EEP may have been partially due to the high antioxidant potential of the propolis extract. Propolis extracts collected from different geographical regions have been characterized by high antioxidant properties, which has been confirmed, among other things, by their DPPH free radical scavenging activities, Fe^3+^-reducing power assays, and ferrous ion (Fe^2+^) chelating activities [56,57,58,61]. The significant increase in the antioxidant activity of the solution with propolis extract and caffeine could be attributed to the presence of phenolic compounds in propolis, including flavonoids and aromatic acids with antioxidant potential [62,63,64,65]. In addition, it was noted that the choice of acid used as a chitosan solvent affected the antioxidant activities of the final solutions. The presented results indicated that the addition of caffeine and propolis extract increased the antiradical effect of the solution based on the chitosan being dissolved in citric acid.

### 3.2. Antimicrobial Activity of the Chitosan-Based Films

In order to evaluate the potential use of the chitosan-based materials for medical applications, it was extremely important to analyze their antimicrobial activity. The results of the antibacterial activities of the chitosan-based films as expressed by the diameters of the inhibition zones are presented in Table 2.

The results indicated that chitosan film prepared with acetic acid as a solvent did not show activities against the tested bacteria strains. In contrast, the chitosan film obtained with citric acid showed bactericidal activities against all tested strains except *S. aureus*. The applied acid also affected the antibacterial activities of chitosan films with caffeine and propolis extract. In the case of the chitosan-based films prepared with acetic acid, their antimicrobial activities were recorded only against selected strains of the pathogenic bacteria. The film that consisted of chitosan and caffeine (sample B) exhibited moderate activity against *E. coli* and *P. aeruginosa*, while the film based on chitosan, caffeine, and EEP (sample C) showed activity against *P*. *aeruginosa* and *S. enterica*. The highest activity was observed for the film obtained from chitosan dissolved in citric acid with the addition of caffeine and propolis extract (sample F). This film showed activities against all the tested bacterial strains (except of *S. aureus*), with diameters in the inhibition zones that ranged from 25 to 28 mm. Also, the film based on chitosan dissolved in citric acid and caffeine showed high antibacterial activity, with zones of inhibition ranging from 19 mm (*S. enterica*) to 28 mm (*E. faecalis* and *E. coli*).

The higher antibacterial activities of the chitosan-based films with the additives compared to the films without them were a consequence of the activities of caffeine and propolis. The literature data have shown that caffeine exhibits antimicrobial activity against *P. fluorescens*, *E. coli,* and *P. aeruginosa* [66,67]. The activities of the propolis extracts against a broad spectrum of bacteria, including gram-positive (*S. aureus*, *B. subtilis*, *B. cereus,* and *E. faecalis)* and gram-negative bacteria (*E*. *coli* and *P. aeruginosa*), have also been confirmed [53,65,68]. The antibacterial effects of the films based on chitosan and propolis extracts have been reported in the literature data. The results described by Siripatrawan and Vitchayakitti [69] showed that propolis extract applied as an additive to chitosan films had a positive effect on their activities against such bacterial strains as *S. aureus*, *Salmonella Enteritidis*, *E. coli,* and *P. aeruginosa*. A study conducted by Stanicka et al. [36] also showed that the addition of EEP improved the activities of chitosan films against *L. monocytogenes*, *S. aureus,* and *B. cereus*. In turn, the films based on chitosan nanoparticles with propolis extracts showed strong inhibitory effects on *L. monocytogenes* and *E. coli* [70].

The obtained chitosan-based films were also tested for their effects on the growth of three strains of probiotic bacteria. The results are presented in Table 3.

It was found that none of the chitosan-based films showed antagonistic activities against the probiotic strains that were tested. Unexpectedly, it was detected that all chitosan-based films stimulated the growth of the probiotic strain *L. plantarum* 299v. In addition, the stimulation of *L. rhamnosus* GG growth was visible when the films produced using citric acid as a chitosan solvent were used. This is an advantageous phenomenon for the potential use of chitosan-based materials as probiotic encapsulation materials. The results obtained were in line with the literature data, which indicated the possibility of using chitosan materials for the encapsulation of live probiotic bacteria, among other things [71,72]. Thus far, the phenomenon of the growth stimulation of probiotic bacteria, even with introduced compounds with antimicrobial activities, has not been described in the literature. This development could have great practical significance. Materials based on chitosan with added caffeine and/or propolis extract used to encapsulate probiotic bacteria could be used, on the one hand, to stimulate their growth, and on the other hand, to protect them from contamination by pathogenic bacteria.

The effects of the obtained chitosan-based films on three strains of fungi were also evaluated, and the results are presented in Table 4.

In the case of the antifungal activities of the tested chitosan-based films, the zone of inhibition against *C. albicans* was observed only for the film based on chitosan, citric acid, caffeine, and EEP (sample F), and it was 22 mm. Research on the antifungal effects of chitosan-based materials with the addition of propolis extract was also conducted by Humelnicu et al. [73]. The results of this study confirmed that these films possessed antifungal activities against *C. albicans* [73]. According to numerous literature data, propolis extracts have antagonistic activities, including against the following yeasts and dermatophytes: *C. albicans*, *Candida tropicalis*, *Cryptococcus neoformans,* and *Microsporum gypseum* [74,75,76]. The literature data have indicated that propolis may affect the integrity of the cell wall of *C. albicans*, and as a result, it exhibits antifungal activity against this strain [77]. A similar observation was reported by Stahli et al. [78], where the ethanol extract of propolis caused a loss in the cell wall integrity of *C. albicans* and a decrease in its metabolic activity. Based on these data, it was found that the activity of the chitosan film detected in this work was the result of the activity of the propolis additive. Moreover, the propolis extracts showed activities against *A. niger* and *A. flavus*, but the fungi were less sensitive or even insensitive to the propolis extracts at lower concentrations compared to the effect of the extracts on bacteria [79,80,81]. In this study, no antifungal activities of any of the chitosan films on *A. niger* were found. Irrespective what acid was used, the films with caffeine and propolis additive limited the growth of *A. flavus*. Even after 21 days of incubation, the plates with such films were not completely overgrown by these molds.

### 3.3. Mechanical Properties

**Figure 3:**
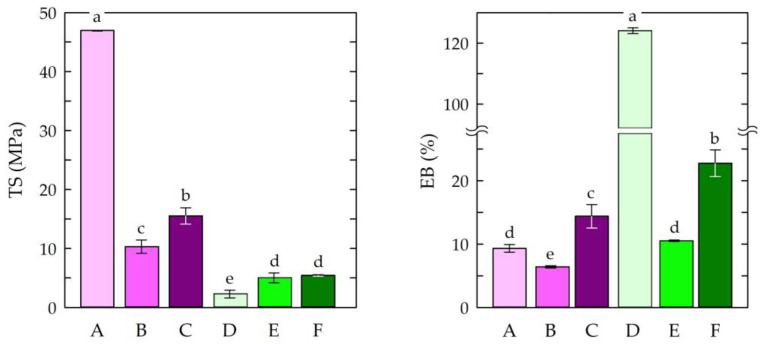
Tensile strength (TS) and elongation at break (EB) measurements of the chitosan film samples. Symbols of chitosan-based films (A–F) according to Table 1. The different letters above the bars in the graph indicate statistically significant differences at *p* < 0.05.

The tensile strength and elongation at break of materials enable them to resist tensile stress and shape changes before breaking [82]. Therefore, the mechanical parameters of the chitosan-based films were determined. Figure 3 presents the tensile strength (TS) and elongation at break (EB) measurements of the obtained films.

The highest tensile strength value (46.95 MPa) was observed for sample A, in which chitosan/acetic acid was used, without the addition of modifiers. The addition of caffeine and EEP resulted in decrease in tensile strength to 10.3 MPa (sample B) and 15.52 MPa (sample C), respectively. It has been established that cross-linking chitosan with citric acid can result in increased water resistance. In addition, the chitosan films cross-linked with citric acid were found to be more resistant to thermal decomposition. Citric acid also exhibits plasticizing effects that can be attributed to its hydroxyl groups and residual carboxyl groups. As a result, the addition of citric acid can significantly improve the elongation of a chitosan film [83]. In this study, the films in which citric acid was used as a solvent showed lower tensile strength values. This was due to the formation of a chemical cross-linking structure [84]. For elongation at break (EB), the highest value was observed for the chitosan–citric acid film (sample D). This resulted from the plasticizing effect of the citrate ions observed in the chitosan–citric acid systems [85]. The other samples had similar elongation values. This was an effect of the modifications used i.e., the addition of caffeine and EEP. This may have been caused by the insufficient dispersion of the modifiers in the chitosan matrices and, thus, the insufficient formation of hydrogen bonds and ionic interactions.

### 3.4. Scanning Electron Microscopy (SEM)

**Figure 4:**
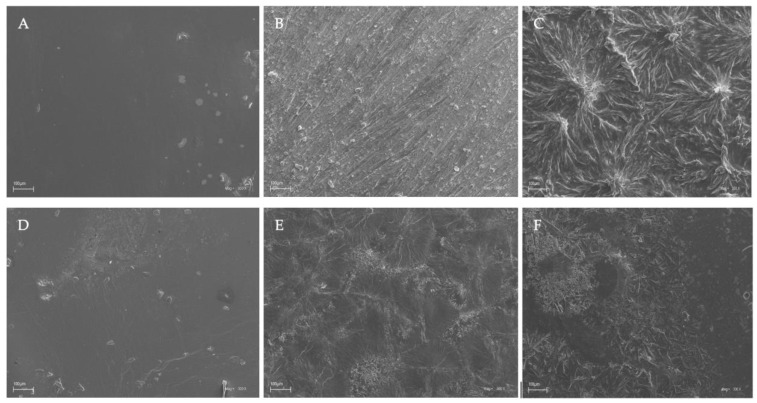
SEM images of the chitosan-based films. Symbols of chitosan-based films (**A**–**F**) according to Table 1.

The surface characteristics of the chitosan-based films were investigated by scanning electron microscopy, and the results of this analysis are shown in Figure 4.

Based on the images obtained using scanning electron microscopy (SEM), it was observed that chitosan films without the additives (caffeine and EEP) in both the acetic and citric acids were characterized by smooth surfaces (Figure 4A,D). This indicated good dispersion of the shrimp chitosan in the solvents that were used. The homogeneous appearances were indicative of the structural integrity of the shrimp chitosan–acid systems used. Only the addition of caffeine and EEP caused irregular clusters of unequal sizes to appear on the surfaces of the prepared films (Figure 4C,F). The clusters formed were the result of the aggregation of caffeine particles on the surfaces. The use of modifications to the chitosan films resulted in their insufficient dispersion in the polymer matrices. In the results, the inadequate formation of hydrogen bonds and ionic interactions, which affected the mechanical properties and the obtained surfaces of the tested samples, could be observed.

### 3.5. Fourier Transform Infrared Spectroscopy (ATR-FTIR)

**Figure 5:**
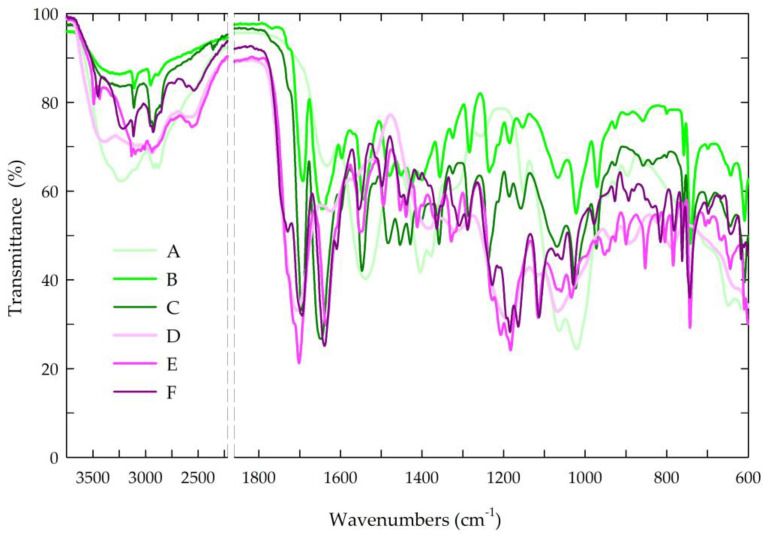
FTIR spectra of the prepared chitosan-based films. Symbols of chitosan-based films (A–F) according to Table 1.

The structures of the chitosan-based films were evaluated by attenuated total reflectance Fourier transform infrared spectroscopy. Figure 5 presents the spectra of all the tested chitosan-based films.

In the spectra of the chitosan films, the bands in the range of 3500–3000 cm^−1^ and at 2880 cm^−1^ corresponded to –OH bonds and aliphatic C–H stretching vibrations [70,86]. Moreover, the FTIR spectra contained bands at 1650 and 1557 cm^−1^, which is characteristic of N-H deformation vibrations coming from the amine groups in amides II and amides III, and the band at 1371 cm^−1^ could be attributed to the C–N bond in amides I. The band at 1025 cm^−1^ indicated C–O stretching in the alcohol group [86].

In the chitosan-based films with caffeine, distinctive bands associated with the caffeine molecule were observed at 1732 cm^−1^ (C=O) and 1650 cm^−1^ (N–H), corresponding to the vibrations of the carbonyl group and the amine groups in the acetamide groups of amide I. In the spectra, the second band of the amine group at 1550 cm^−1^ indicated the stretching vibrations of the N–H and C–N groups coming from amides II (C–N–H) [70,87,88,89]. The broad band of the medium intensity at 765 cm^−1^ indicated the vibrations of the N-H bond.

In the spectra of the chitosan-based films with caffeine and propolis extracts, characteristic bands originating from the propolis, particularly the phenolic compounds, were observed. The bands at 2924 and 2850 cm^−1^ represented the stretching vibrations of the C–H bond, confirming the presence of long-chain alkyl compounds in the propolis extract. The band at 1732 cm^−1^ was characteristic of the stretching vibrations of the carbonyl group (C=O), while the stretching and bending bands at 1634 cm^−1^ were characteristic of the C–O bond. In the spectra, the bands at 1515 and 1451 cm^−1^ (C=C) were mainly associated with the aromatic rings of the phenolic compounds. Additionally, a broad band at approximately 3420 cm^−1^ indicated the stretching vibrations of the O–H bond, further confirming the presence of the phenolic compounds from the propolis [70,90,91]. The observed shifting and/or broadening of certain bonds in the FTIR spectra suggested the existence of intermolecular interactions between the hydroxyl and amine groups in the chitosan and the phenolic compounds in the propolis, aligning with the existing literature [69,70,86].

## 4. Conclusions

This paper presents the results of study of the biological, mechanical, and structural properties of films made from chitosan using two solvents (acetic and citric acid) and natural biologically active substances (caffeine and propolis extract). The addition of caffeine and propolis to chitosan dissolved in citric acid increased the antioxidant activities of the solution. Moreover, the film obtained from this solution showed good antibacterial activity. In a comparison of the antimicrobial activities against pathogenic strains, it was found that the films obtained using citric acid were characterized by higher antimicrobial activities than the films produced with acetic acid. Additionally, they acted against a wider spectrum of bacterial strains. The antibacterial effects of the chitosan-based films were further enhanced by the addition of caffeine and propolis extract. All the tested chitosan-based films showed no activities against probiotic bacterial strains; moreover, they contributed to the stimulation of bacterial growth. The addition of caffeine and propolis extract to the chitosan–acetic acid film caused decreases in the tensile strengths of the films. In the case of the chitosan–citric acid films, the addition of bioactive substances increased the tensile strengths of the films. The obtained results showed that both the addition of bioactive substances (caffeine and propolis extract) and the solvent used to dissolve the chitosan (acetic or citric acid) affected the antioxidant properties of the film-forming solutions as well as the biological and mechanical properties of the obtained films.

The presented results suggested that chitosan-based films with natural bioactive substances are a promising alternative to the traditional materials used in the medical industry, mainly as biodegradable wound dressings. They can also be useful for encapsulating probiotic bacteria.

## Figures and Tables

**Table 1:** The symbols of the chitosan-based film samples.

Symbol	Solvent	Additives	
		Caffeine	Propolis Extract
A	Acetic acid	--	--
B	Acetic acid	✓	--
C	Acetic acid	✓	✓
D	Citric acid	--	--
E	Citric acid	✓	--
F	Citric acid	✓	✓

**Table 2:** The activities of the chitosan-based films against the pathogenic strains of bacteria.

Bacterial Strain	Zone of Inhibition (mm)					
	A	B	C	D	E	F
*B. subtilis*	--	--	--	20	24	26
*E. faecalis*	--	--	--	28	28	28
*E. coli*	--	18	--	22	28	28
*P. aeruginosa*	--	18	18	20	25	27
*P. fluorescens*	--	--	--	24	24	26
*S. enterica*	--	--	16	16	19	25
*S. aureus*	--	--	--	--	--	--

**Table 3:** The activities of the chitosan-based films against probiotic bacteria strains.

Bacterial Strain	Activity					
	A	B	C	D	E	F
*L. paracasei*	--	--	--	--	--	--
*L. rhamnosus*	--	--	--	S	S	S
*L. plantarum*	S	S	S	S	S	S

S, stimulation of bacteria growth.

**Table 4:** The activities of the chitosan-based films against fungal strains.

Fungal Strain	Activity					
	A	B	C	D	E	F
*A. flavus*	--	-- ^	-- ^	--	-- ^	-- ^
*A. niger*	--	--	--	--	--	--
*C. albicans*	--	--	--	--	--	22 * st

st, static action; ^, limitation of mold growth (the mold did not grow all over the plate); *, inhibition zone (mm).

## Data Availability

Not applicable.
